# Elevation of tacrolimus concentration after administration of methotrexate for treatment of graft-versus-host disease in pediatric patients received allogeneic hematopoietic stem cell transplantation

**DOI:** 10.1186/s40780-023-00306-w

**Published:** 2023-12-05

**Authors:** Chiaki Inoue, Takehito Yamamoto, Hiroshi Miyata, Hiroshi Suzuki, Tappei Takada

**Affiliations:** 1 Department of Pharmacy, The University of Tokyo Hospital, Faculty of Medicine, The University of Tokyo, 7-3-1 Hongo, Bunkyo-Ku, Tokyo, 113-8655 Japan; 2 The Education Center for Clinical Pharmacy, Graduate School of Pharmaceutical Sciences, The University of Tokyo, 7-3-1 Hongo, Bunkyo-Ku, Tokyo, 113-0033 Japan

**Keywords:** Tacrolimus, Methotrexate, Hematopoietic stem cell transplantation, Therapeutic drug monitoring

## Abstract

**Background:**

Methotrexate (MTX) is used to treat graft-versus-host disease (GVHD) following allogeneic hematopoietic stem cell transplantation (allo-HSCT). Recently, a case was encountered in which the blood concentration of tacrolimus (TCR) at steady state increased after intravenous MTX administration for GVHD treatment (therapeutic IV-MTX administration). Therefore, this study aimed to investigate the effect of therapeutic IV-MTX administration on the pharmacokinetics of TCR.

**Methods:**

This single-center, retrospective, observational study included patients who underwent allo-HSCT and received therapeutic IV-MTX administration during immunosuppressive therapy with continuous intravenous infusion (CIV) of TCR from April 2004 to December 2021. Here, each therapeutic IV-MTX administration was defined as a case and independently subjected to subsequent analyses. The blood concentration of TCR at steady state (C_ss_), ratio of C_ss_ to daily TCR dose (C/D), and clinical laboratory data were compared before and after therapeutic IV-MTX administration. In addition, dose changes in the TCR after therapeutic IV-MTX administration were evaluated.

**Results:**

Ten patients (23 cases) were included in this study. The C/D value significantly increased after therapeutic IV-MTX administration (median: 697 vs. 771 (ng/mL)/(mg/kg), 1.16-fold increase, *P* < 0.05), indicating a reduction in the apparent clearance of TCR. Along with the increase in C/D, significant increases were observed in aspartate transaminase level (median: 51.0 vs. 92.9 U/L, *P* < 0.01) and alanine aminotransferase level (median: 74.5 vs. 99.4 U/L, *P* < 0.01) indicating that liver injury after therapeutic IV-MTX administration contributes to the observed C/D increase. In addition, the daily dose of TCR was reduced in 11 cases (47.8%) after therapeutic IV-MTX administration, and the relative frequency of dose reduction tended to be higher than that of dose increase (median: 37.5% vs. 0.0%, *P* = 0.0519, permuted Brunner-Munzel test). The magnitude of dose reduction was 18.8% (7.4–50.0%) in the 11 cases with dose reduction.

**Conclusions:**

Therapeutic IV-MTX administration cause a significant increase in C/D, which requires TCR dose reduction. Careful therapeutic drug monitoring of TCR is needed after therapeutic IV-MTX administration in patients receiving immunosuppressive therapy with TCR after allo-HSCT.

## Background

Tacrolimus (TCR), a calcineurin inhibitor, is widely used for graft-versus-host disease (GVHD) prophylaxis after allogeneic hematopoietic stem cell transplantation (allo-HSCT). Previous studies have demonstrated that the clinical efficacy and toxicity of TCR are correlated with its blood concentration, low concentration is associated with insufficient immunosuppression, which may lead to GVHD, whereas high concentration increases the risk of adverse reactions such as kidney injury and encephalopathy [1–3]. Owing to its narrow therapeutic range and large inter- and intra-patient variability in pharmacokinetics (PK) [4], dose adjustment based on therapeutic drug monitoring (TDM) is important during immunosuppressive therapy with TCR. As TCR is mainly metabolized by cytochrome P450 (CYP) 3A, numerous studies have reported the alterations in the PK of TCR via CYP3A mediated drug-drug interactions (DDIs). However, limited information is available regarding the impact of the concomitant administration of drugs other than typical CYP3A inhibitors/inducers on the PK of TCR.

**Fig. 1:**
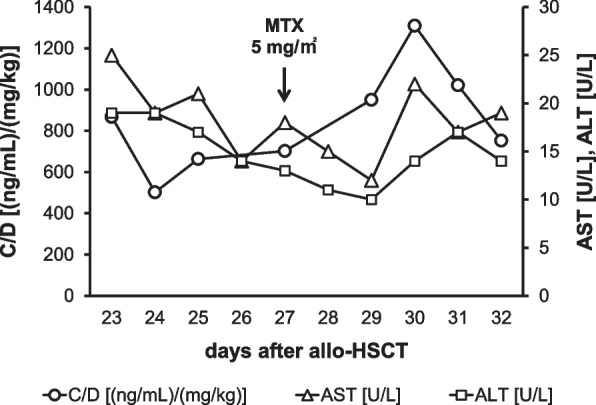
The increase in C/D of the patient after therapeutic IV-MTX administration

Methotrexate (MTX), a folate metabolism inhibitor, is frequently administered concomitantly with TCR for GVHD prophylaxis and/or treatment [5–9]. Recently, a pediatric case exhibiting elevated blood concentrations of TCR at steady state (C_ss_) after intravenous MTX administration for GVHD treatment (hereafter referred to as “therapeutic IV-MTX administration”) was encountered. The patient had received TCR as continuous intravenous infusion (CIV) from the day before allo-HSCT (day -1) and MTX as intravenous injection (IV) in doses of 10, 7, and 7 mg/m^2^ on days 1, 3, and 6, respectively, for GVHD prophylaxis. On day 27, the patient developed acute GVHD and received therapeutic IV-MTX administration (5 mg/m^2^) after which, C_ss_ started to increase, and the ratio of C_ss_ to the daily TCR dose (C/D), a surrogate marker of TCR clearance, showed an increasing trend until day 30 (Fig. 1). Considering that TCR was consistently administered as CIV until day 33, the observed increase in C/D indicated the decrease in systemic clearance of TCR, not the increase in bioavailability.

However, because MTX is unlikely to inhibit CYP3A enzymes, this increase in the systemic clearance of TCR would not be attributable to DDI via alteration of CYP3A activity. This encouraged a further investigation of the possible effects of therapeutic IV-MTX administration on the PK of TCR.

To elucidate the effect of therapeutic IV-MTX administration on the PK of TCR in patients who underwent allo-HSCT, the changes in C/D before and after therapeutic IV-MTX administration for GVHD were retrospectively investigated along with the resulting TCR dose changes after therapeutic IV-MTX administration.

## Methods

### Study design and patients

This single-center, retrospective, observational study was conducted at The University of Tokyo Hospital (Tokyo, Japan). Patients who underwent allo-HSCT and received therapeutic IV-MTX administration during immunosuppressive therapy with TCR (administered as CIV) from April 2004 to December 2021 were assessed for eligibility. Each therapeutic IV-MTX administration was defined as a case, when a patient received therapeutic IV-MTX administration more than once during the study period, each case was independently assessed for eligibility. When conducting the PK analysis, the cases that met the following exclusion criteria were excluded: 1) oral TCR was administered between 2 days before and 4 days after the therapeutic IV-MTX administration, 2) TCR CIV administration was suspended between 2 days before and 4 days after the therapeutic IV-MTX administration due to steroid pulse therapy for acute GVHD, 3) MTX was re-administered within 4 days after the previous therapeutic IV-MTX administration because it was difficult to judge which therapeutic IV-MTX administration affected C/D, 4) C_ss_ was not measured before (on the day or one day before the day) and/or after (from 2 to 4 days after) the therapeutic IV-MTX administration, 5) azole antifungals were newly started or stopped between 2 days before and 4 days after the therapeutic IV-MTX administration, 6) the kind of azole antifungals was switched to another azole antifungal between 2 days before and 4 days after the therapeutic IV-MTX administration, and 7) aprepitant, well-known CYP3A inhibitor and inducer [10, 11] was administered within 3 weeks before therapeutic IV-MTX administration.

### Treatments

Allo-HSCT was performed after conditioning pretreatment with chemo(radio)therapy in The University of Tokyo Hospital (Tokyo, Japan), following which, immunosuppressive treatment with TCR and MTX was started to prevent GVHD [5, 6]. TCR was generally started from the day before allo-HSCT (day -1) at a dose of 0.02 mg/kg/day CIV and the dose was adjusted at the discretion of the attending physician based on TDM and clinical condition. MTX was administered intravenously on days 1, 3, 6, and 11 at doses of 15, 10, 10, and 10 mg/m^2^, respectively, or on days 1, 3, and 6 at doses of 10, 7, and 7 mg/m^2^, respectively.

The therapeutic IV-MTX was administered to the patients who developed GVHD at a dose of 3–10 mg/m^2^ weekly until the GVHD resolved [7–9].

### Data collection

The following demographic and clinical data were collected using the electronic medical record system: age at the time of allo-HSCT, sex, body weight, primary disease, conditioning pretreatment, classification of allo-HSCT, the doses of TCR and MTX, C_ss_, concomitant use of azole antifungals and/or aprepitant, red blood cell (RBC) count, hematocrit (Hct), hemoglobin (Hb), serum albumin (Alb), aspartate aminotransferase (AST), alanine aminotransferase (ALT), total bilirubin (T-bil), and serum creatinine (Cre) levels. In the hospital, blood concentrations of TCR were measured using chemiluminescent immunoassay (CLIA, ARCHITECT Tacrolimus assay, Abbott) (~ June 13^th^, 2021) and affinity column-mediated immune assay (ACMIA, Dimension Tacrolimus Flex reagent cartridge, Siemens Healthineers) (June 14^th^, 2021 ~).

Clinical laboratory data were obtained on the same day as C_ss_ measurements. For clinical laboratory data after MTX administration, average values from 2 to 4 days after therapeutic IV-MTX administration were used. When more than one case of therapeutic IV-MTX administration was eligible in a single patient, the mean value of clinical data of all cases in the patient was used in the analysis of changes in clinical laboratory data after therapeutic IV-MTX administration.

### Calculation of C/D

The C/D was calculated by the following equation [12]:

$$C/D [(ng/mL)/(mg/kg)]=\frac{{C}_{ss}\left[ng/mL\right]}{Daily TCR dose \left[mg/kg\right]}$$

In this equation, the daily dose of TCR on the day before the C_ss_ measurement was used. The C/D values were calculated before and after therapeutic IV-MTX administration to evaluate its effects on the clearance of TCR. The C/D before therapeutic IV-MTX administration was calculated using the C_ss_ measured immediately before (or measured on the previous day when C_ss_ on the day of MTX administration was not measured). In contrast, the mean C/D value from days 2 to 4 after therapeutic IV-MTX administration was used as the C/D value after therapeutic IV-MTX administration. When more than one case of therapeutic IV-MTX administration was eligible in a single patient, the mean value of C/D and fold changes in C/D of all cases in the patient were used in the analysis of changes in C/D after therapeutic IV-MTX administration.

### Evaluation of dose changes of TCR after therapeutic IV-MTX administration

The daily doses of TCR from the next day to 7 days after therapeutic IV-MTX administration were compared with those on the day of MTX administration to investigate changes in TCR doses. Dose changes were classified into three types: dose reduction, dose increase, and no change compared with the previous day. When both dose reduction and increase occurred from the next day to 7 days after therapeutic IV-MTX administration, the first change in the TCR dose was analyzed. When the TCR doses did not change from the next day to 7 days after therapeutic IV-MTX administration, these cases were classified as “no change”.

### Statistical analysis

Continuous variables are shown as median (range) unless otherwise stated. Because the number of cases of therapeutic IV-MTX administration differed among patients, the geometric mean values of C/D before and after therapeutic IV-MTX administration in each case within the same patient were subjected to the following statistical analysis. Similarly, the relative frequency of each type of dose change (dose reduction, dose increase, or no change) for each patient was calculated. Wilcoxon signed-rank tests were used to compare the C/D and clinical laboratory data before and after therapeutic IV-MTX administration. A permuted Brunner-Munzel test was performed to compare the relative frequencies of each type of dose change [13].

## Results

### Characteristics of patients

**Fig. 2:**
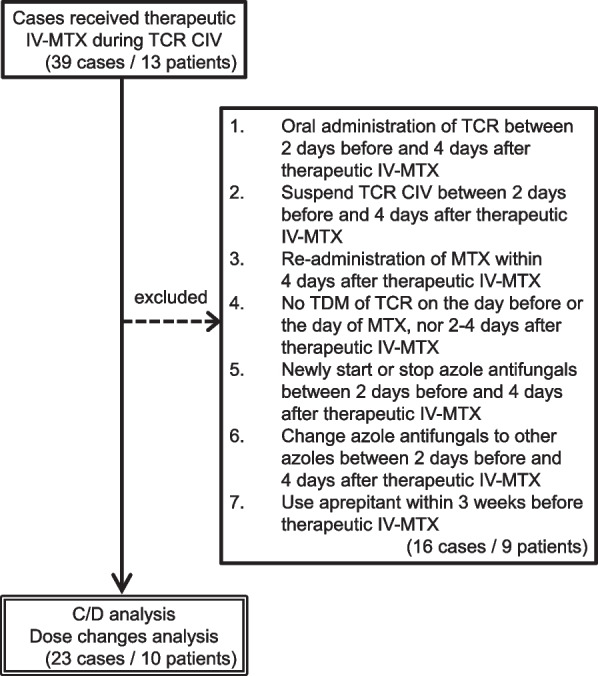
Selection flow of cases in this study

Thirteen patients (39 cases) were assessed for eligibility and 10 patients (23 cases) were included in the subsequent analysis (Fig. 2). Table 1 shows the demographic and clinical characteristics of the patients. As shown in Table 1, all patients were pediatric (< 12 years old), and 6 out of 10 patients received cord blood transplantation (CBT). Although one patient received voriconazole at a stable dose, there were no cases in which azole antifungals were newly started, stopped, or changed between 2 days before and 4 days after therapeutic IV-MTX administration. Five patients received aprepitant; however, more than 3 weeks had passed before therapeutic IV-MTX administration in all five patients. The grades of GVHD in our study population were: Grade I, 5 patients; Grade I–II, 1 patient (skin GVHD stage 1 and gut GVHD stage 0–1); Grade II, 2 patients; and unknown, 2 patients. Only one patient was diagnosed with hepatic GVHD. No drugs other than MTX that may cause liver injury (i.e., voriconazole or acetaminophen) were newly started.

**Table 1:** Demographic and clinical characteristics of patients

Patient No	Age [years]	Sex	BW [kg]	Primary disease	Conditioning pretreatment	Type of HSCT	RBC [× 10^4^/μL]	Hct [%]	Hb [g/dL]	Alb [g/dL]	AST [U/L]	ALT [U/L]	T-bil [mg/dL]	Cre [mg/dL]
#1	4.3	M	13.3	CGD	ATG, BU, Flu, TBI	rPBSCT	332	28.9	9.9	3.8	43	121	0.3	0.18
#2	6.8	F	22.0	BCP-ALL	Mel, TBI	CBT	297	26.7	9.1	3.5	51	56	0.5	0.29
#3	9.6	F	35.2	AML	CY, TBI	CBT	290	26.2	8.9	3.3	76	89	0.8	0.24
#4	7.8	M	18.2	AML	BU, Mel	rPBSCT	312	32.5	10.7	2.6	113	150	0.5	0.23
#5	6.8	M	29.8	MDS	ATG, Flu, Mel, TBI	uBMT	348	33.1	11.5	3.6	51	109	1.2	0.32
#6	11.4	F	25.3	T-ALL	CY, VP-16, TBI	uBMT	287	28.2	9.6	2.4	96	95	3.5	0.24
#7	2.3	F	11.3	AML	BU, CY	CBT	315	33.2	10.9	3.2	27	47	0.4	0.13
#8	4.6	M	16.0	BCP-ALL	CY, VP-16, TBI	CBT	297	27.4	9.0	4.4	26	34	1.0	0.20
#9	2.6	F	9.8	EBV-LPD	Ara-C, Flu, Mel, MIT, VP-16, PSL	CBT	334	28.5	10.1	3.5	18	13	3.3	0.21
#10	0.7	F	7.3	CAMT	ATG, Flu, Mel	CBT	417	34.3	12.3	3.8	67	60	0.6	0.25
Median (range)	5.7 (0.7–11.4)		17.1 (7.3–35.2)				314 (287–417)	28.7 (26.2–34.3)	10.0 (8.9–12.3)	3.5 (2.4–4.4)	51 (18–113)	75 (13–150)	0.7 (0.3–3.5)	0.23 (0.13–0.32)

*M* Male, *F* Female, *BW* Body weight, *CGD* Chronic granulomatous disease, *BCP-ALL* B-cell precursor acute lymphoblastic leukemia, *AML* Acute myeloid leukemia, *MDS* Myelodysplastic syndromes, *T-ALL* T-cell acute lymphoblastic leukemia, *EBV-LPD* Epstein-Barr virus-associated lymphoproliferative disease, *CAMT* Congenital amegakaryocytic thrombocytopenia, *HSCT* Hematopoietic stem cell transplantation, *ATG* Anti-thymocyte globulin, *BU* Busulfan, *Flu* Fludarabine, *TBI* Total body irradiation, *Mel* Melphalan, *CY* Cyclophosphamide, *VP-16* Etoposide, *Ara-C* Cytarabine, *MIT* Mitoxantrone, *PSL* Prednisolone, *rPBSCT* Related peripheral blood stem cell transplantation, *CBT* Cord blood transplantation, *uBMT* Unrelated bone marrow transplantation, *RBC* Red blood cell, *Hct* Hematocrit, *Hb* Hemoglobin, *Alb* Albumin, *AST* Aspartate aminotransferase, *ALT* Alanine transferase, *T-bil* Total bilirubin, *Cre* Serum creatinine

### Changes in C/D after therapeutic IV-MTX administration

**Fig. 3:**
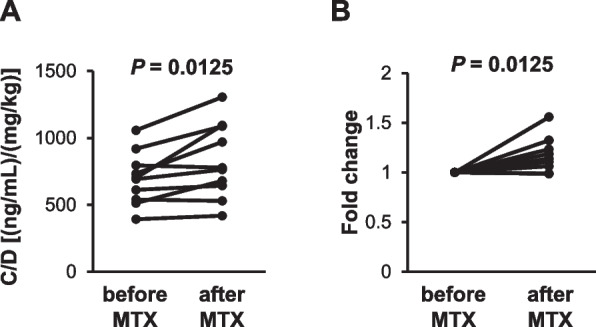
Changes in TCR C/D after therapeutic IV-MTX administration. Absolute values of C/D [(ng/mL)/(mg/kg)] (**A**) and fold changes in C/D before and after therapeutic IV-MTX administration (**B**) are shown. Each plot represents individual data. *P* values were determined using the Wilcoxon signed-rank test

The measurement method of TCR was not changed before or after therapeutic IV-MTX administration in all cases. The infusion rate of TCR was constant in all the patients for more than 24 h before TCR blood concentration measurement, which enables us to employ C/D as the reliable index of TCR clearance. As shown in Fig. 3A, C/D significantly increased after MTX administration (697 vs. 771 [(ng/mL)/(mg/kg)] before vs. after therapeutic IV-MTX administration, respectively; *P* = 0.013, Wilcoxon signed-rank test). The relative magnitude of increase after MTX administration was 1.16-fold (0.99–1.56) (*P* = 0.013, Wilcoxon signed-rank test) (Fig. 3B).

### Changes in clinical laboratory data after therapeutic IV-MTX administration

**Fig. 4:**
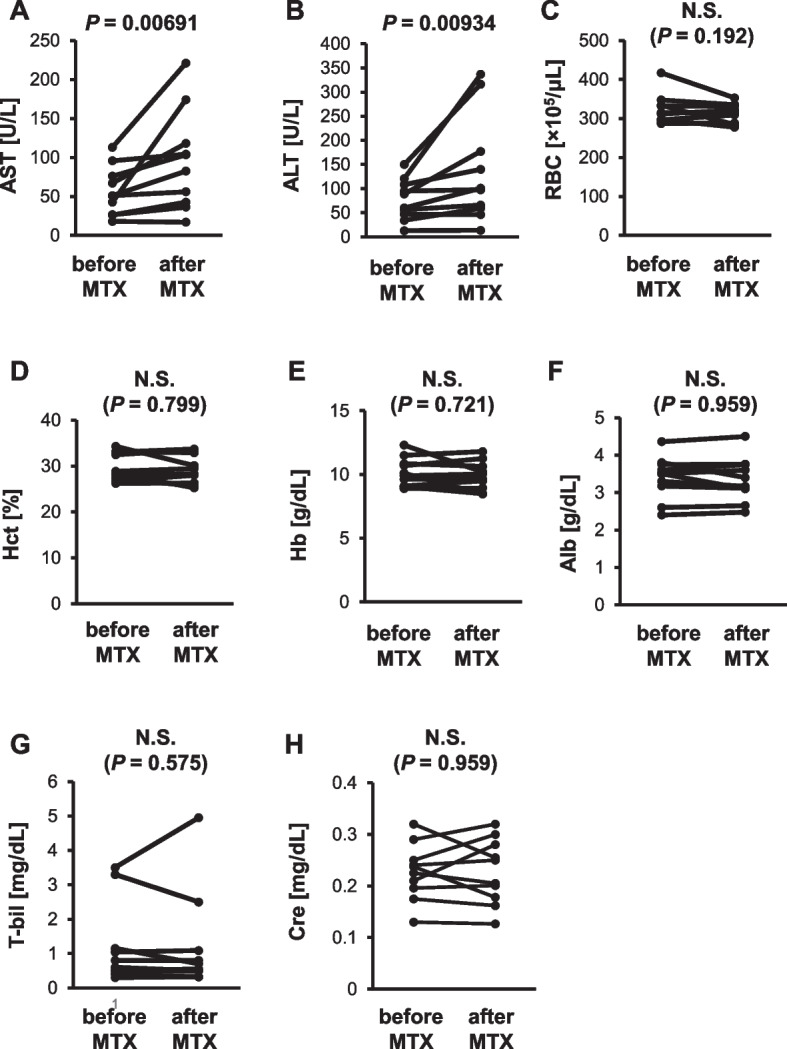
Changes in clinical laboratory data after therapeutic IV-MTX administration. Changes in AST (**A**) and ALT (**B**) levels, RBC count (**C**), Hct (**D**), Hb (**E**), Alb (**F**), T-bil (**G**), and Cre (**H**) levels after therapeutic IV-MTX administration are shown.* P* values were determined using the Wilcoxon signed-rank test. AST, aspartate aminotransferase; ALT, alanine transferase; RBC, red blood cell; Hct, hematocrit; Hb, hemoglobin; Alb, albumin; T-bil, total bilirubin; Cre, serum creatinine

To explore the mechanism underlying the increase in C/D after therapeutic IV-MTX administration, the changes in the clinical laboratory data of the patients were investigated. As shown in Fig. 4, statistically significant increases were observed in AST level (51.0 vs. 92.9 U/L, *P* < 0.01) and ALT level (74.5 vs. 99.4 U/L, *P* < 0.01) (Fig. 4A, B). In contrast, there were no significant differences in RBC count, Hct, Hb, Alb, T-bil, and Cre levels before and after therapeutic IV-MTX administration (Fig. 4C–H).

### Dose changes in TCR after therapeutic IV-MTX administration

Whether TCR doses changed in response to changes in C/D after therapeutic IV-MTX administration was also investigated. Among the 23 cases analyzed, the TCR doses decreased and increased in 11 and 3 cases, respectively, after MTX administration (Table 2). The relative frequency of dose reduction was numerically higher than that of dose increase (37.5% vs. 0.0%, *P* = 0.0519, permuted Brunner-Munzel test). In addition, the magnitude of dose reduction was 18.8% (7.4–50.0%) in cases with dose reduction.

**Table 2:** Dose changes in TCR after therapeutic IV-MTX administration

Patient No	Total cases	Reduction cases (%)	Increase cases (%)	No change cases (%)
#1	2	0 (0)	1 (50)	1 (50)
#2	1	1 (100)	0 (0)	0 (0)
#3	1	0 (0)	0 (0)	1 (100)
#4	2	0 (0)	0 (0)	2 (100)
#5	2	1 (50)	0 (0)	1 (50)
#6	4	3 (75)	0 (0)	1 (25)
#7	4	1 (25)	2 (50)	1 (25)
#8	5	4 (80)	0 (0)	1 (20)
#9	1	1 (100)	0 (0)	0 (0)
#10	1	0 (0)	0 (0)	1 (100)

## Discussion

This study provides two important findings: 1) C/D increased after therapeutic IV-MTX administration which required TCR dose reduction, and 2) AST and ALT levels were significantly increased along with the increase in C/D, indicating that injured liver function after therapeutic IV-MTX administration is one of the mechanisms underlying the increase in C/D. These findings indicate the need for careful monitoring of TCR blood concentration after therapeutic IV-MTX administration.

The C/D significantly increased after therapeutic IV-MTX administration (Fig. 3). To the best of our knowledge, this is the first report demonstrating an increase in the C/D of TCR after therapeutic IV-MTX administration. Because all C/D values were calculated under TCR CIV administration in this study, the observed increase in C/D is attributable to the decrease in systemic clearance of TCR, not the alteration in bioavailability. In addition, patients who had recently started or stopped azole antifungals or switched azole antifungals to another azole were excluded to eliminate the effects of CYP3A inhibition by azole antifungals. Patients who received aprepitant were also excluded because, in addition to its inhibitory effect on CYP3A, it is known to induce CYP3A for approximately three weeks after discontinuation of its use. Considering that MTX is unlikely to inhibit or induce CYP3A, the observed decrease in TCR clearance after therapeutic IV-MTX administration is unlikely to be attributable to a DDI via CYP3A.

To explore the underlying mechanisms for the increase in C/D after therapeutic IV-MTX administration, the clinical laboratory data of patients before and after therapeutic IV-MTX administration were compared because some clinical laboratory data have been reported to be associated with changes in TCR C/D [14–20]. It was found that AST and ALT levels significantly increased after therapeutic IV-MTX administration, whereas RBC count, Hct, Hb, Alb, T-bil, and Cre levels, those factors reportedly affect the C/D in previous studies [14–16, 18–20], were not significantly different before and after therapeutic IV-MTX administration (Fig. 4). Liver injury, represented by a transient or prolonged increase in AST and ALT levels is one of the adverse effects of MTX [21] and is reportedly observed in approximately 50% of patients who received it [22]. Because TCR is mainly eliminated from the body via metabolism by hepatic CYP3A, the increase in AST and ALT levels after MTX administration may explain the decreased clearance of TCR.

Severe GVHD (Grades III or IV) was also shown as a covariate of TCR clearance [14], while the effects of moderate GVHD (Grades I or II) on TCR clearance were not examined. Thus, although whether moderate GVHD affects TCR clearance is unknown, the magnitude of the effect would be weaker than that observed in patients with severe GVHD even if moderate GVHD affects TCR clearance. In this study, 8 or more out of the 10 patients developed moderate GVHD. Therefore, we speculated that the onset of GVHD had a limited impact on TCR C/D. In addition, TCR C/D reportedly increased after red blood cell transfusion along with the increase in the hematocrit levels before and after transfusion [23]. In this study, although 9 of 10 patients received red blood cell transfusions during the observation period, no significant change in hematocrit levels was observed before and after therapeutic IV-MTX administration. Therefore, we speculate that the impact of red blood cell transfusions on TCR C/D is limited in our study population.

The relative frequency of dose reduction was numerically higher than that of dose increase (Table 2). In addition, the magnitude of dose reduction was 18.8% (7.4–50.0%) in the 11 cases with dose reduction. These results indicate that changes in TCR clearance after therapeutic IV-MTX administration have a clinical impact on the dose adjustment of TCR in clinical settings. Because appropriate management of TCR blood concentration is critical, especially in GVHD treatment, it is important to note that therapeutic IV-MTX administration may require a dose reduction of TCR. When MTX is administered for GVHD treatment, careful attention should be paid to fluctuations in liver function and TCR blood concentrations, and frequent TDM is considered necessary.

This study has several limitations that need to be considered. First, this was a single-center, retrospective, observational study with a small number of pediatric patients. Therefore, the generalizability of this study should be confirmed in future studies. The results of this study should be carefully extrapolated especially to adult patients. Second, the timing of C/D evaluation varied in this study. Hence, we cannot rule out the possibility that variation in the timing of the C/D evaluation may have contributed to the differences in the magnitude of the changes in C/D observed among the patients in this study. To assess the changes in C/D induced by MTX more quantitatively and precisely, further studies in which the timing of C/D evaluation is unified are needed. Third, the duration of the effect of therapeutic IV-MTX administration on TCR C/D was not investigated in this study. Although we speculate that the effect of therapeutic IV-MTX is temporal and TCR C/D would return to baseline within one week based on the observation in the case shown in Fig. 1, future studies are needed to elucidate the duration of the effect of therapeutic IV-MTX administration. Fourth, the focus was on only MTX administration for the treatment, not the prevention, of GVHD, because the timing of MTX administration for the prevention was when TCR CIV had just been started and TCR blood concentration had not stabilized. Therefore, whether an increase in C/D occurs only in the case of MTX administration for GVHD treatment or in any other case of MTX administration remains to be investigated.

## Conclusion

This study revealed that C/D increased significantly after therapeutic IV-MTX administration, requiring dose adjustment of TCR. The results of this study would contribute to the precise management of TCR concentrations in pediatric patients who received allo-HSCT.

## Data Availability

The datasets used and/or analyzed during the current study are available from the corresponding author on reasonable request.

## Abbreviations

Alb: Albumin
allo-HSCT: Allogeneic hematopoietic stem cell transplantation
ALT: Alanine aminotransferase
AST: Aspartate aminotransferase
C/D: Concentration per dose
CIV: Continuous intravenous infusion
Cre: Serum creatinine
C_ss_: Blood concentration of tacrolimus at steady state
CYP: Cytochrome P450
DDI: Drug-drug interaction
GVHD: Graft-versus-host disease
Hb: Hemoglobin
Hct: Hematocrit
IV: Intravenous injection
MTX: Methotrexate
PK: Pharmacokinetics
RBC: Red blood cell
T-bil: Total bilirubin
TCR: Tacrolimus
TDM: Therapeutic drug monitoring
